# Workplace Resocialization After Parental Leave as a Site of Work/Life Paradox in Three Boundary-Setting Contexts

**DOI:** 10.3390/bs15091224

**Published:** 2025-09-09

**Authors:** Emily A. Godager, Sarah E. Riforgiate

**Affiliations:** 1 Independent Scholar, Milwaukee, WI 53211, USA; 2 Department of Communication, University of Wisconsin-Milwaukee, Milwaukee, WI 53211, USA

**Keywords:** organizational resocialization, organizational assimilation, organizational individualization, work/life boundary-setting, parental leave, paradox, identity, control/resistance, dialectical tensions

## Abstract

This study attends to employees’ boundary-setting enactments during workplace resocialization following parental leave in the United States. We qualitatively analyzed the work/life boundary-setting enactments of 16 employees who returned to the workplace following parental leave using the dialectical lens of control (organizational assimilation) and resistance (individualization). Findings illustrate how employees managed tensions that generated an overarching work/life paradox during organizational resocialization across identity, time, and topic boundary-setting stressors. Employees’ tensioned enactments illustrated a control/resistance dialectic whereby paradoxical responses (vacillating, integrating, and/or balancing) were used to align with professional norms or privilege a working parent identity. This study contributes to paradox research and the model of organizational socialization to deepen our theoretical understanding of how resocialization is a communication process where managing work/life paradoxical responses to identity, time, and topic stressors can attenuate dialectical organizational tensions. Furthermore, we offer practical recommendations for organizations, supervisors, and individuals to productively understand and approach tensions.

## 1. Introduction

Numerous work and life circumstances require employees to take temporary leave and later re-enter the workplace. Leaves are more common than one might expect and coincide with numerous events, including interim appointments, temporary unit transfers, special projects, satellite work, military leave, extended illness, caring for family, and more. Temporary leaves and subsequent workplace re-entries result in socializing communication which impacts employee work/life negotiations, identities, and organizational processes. While organizational socialization research predominantly focuses on newcomer socialization experiences to learn organizational norms and processes (Riforgiate & Kramer, 2021), socialization is continual throughout one’s career (Gardner et al., 2022). Thus, established employees “may experience many *resocialization* processes throughout their tenure as their role and the organization changes” (Hart et al., 2009, p. 492; emphasis added), particularly when temporary leaves challenge elements of employees’ work/life identities (Ladge & Greenberg, 2015). While socialization research often regards communication as contained within an organization (Kramer, 2010), this study’s focus on re-entry after a temporary leave demonstrates the flexible and permeable boundaries of organizations and illustrates how socialization is an ongoing negotiation of individuals’ experiences that span the work/life boundary.

A common workplace leave and re-entry occurs for childbirth or adoption. Considering the United States context, according to the National Center for Health Statistics, 3,591,328 births occurred in 2023 (Hamilton et al., 2024), affecting millions of parents and the organizations they work for. A quarter of individuals using the United States Family Medical Leave Act do so for childbirth or adoption, only second to personal health issues (Brown et al., 2020). In addition to prevalence, parental leave is a critical transition point that “sets the stage for how [parents] will manage work/life integration for the next phase of [their] career” (Ladge & Greenberg, 2015, p. 977).

However, communicatively (re)negotiating work/life boundaries during re-entry presents challenges due to disparate norms^1^—tensions—that shape new and ongoing enactments of professional and parent identities (Ladge & Greenberg, 2015). This is particularly true in the United States, one of few countries that does not have a national paid family leave policy (Schulte et al., 2017). While formal organizational policies exist to support working parents, deeply entrenched ideal worker and ideal parent norms influence how policies are “interpreted and implemented by supervisors, and their use is influenced by coworkers and the culture related to work/life” (Kirby & Buzzanell, 2014, p. 356). Resocialization experiences exemplify dialectics, competing poles/forces, and related tensions that occur when organizational members’ attempt to shape employees’ behaviors and identity *and* when individuals communicate to reciprocally shape their role or organization’s culture (organizational assimilation and individualization; Kramer & Dailey, 2019).

Approaching assimilation/individualization processes as dialectical during resocialization, we draw on scholarship that has “integrated dialectics with a paradox perspective by casting the former as the generative motor of the latter” (Fairhurst & Putnam, 2024, p. 5). *Paradoxes* occur when “in the pursuit of one goal, the pursuit of another competing goal enters the situation (often without intention) so as to undermine the first pursuit” (Stohl & Cheney, 2001, p. 354). Paradoxical experiences represent “one moment in time within a never-ending dialectical process” which shed light on “persistent contradictions that impose and reflect back on one another to create a seemingly impossible choice” (Fairhurst & Putnam, 2024, p. 16). The paradox of simultaneously fulfilling worker and parent (life) roles following parental leave often illuminates dialectical tensions.

*Dialectics* are the “interdependent opposites aligned with forces that push-pull on each other like a rubber band and exist in an ongoing dynamic interplay as the poles implicate each other” (Putnam et al., 2016, p. 71). We approach assimilation and individualization during parents’ resocialization as a dialectical negotiation process of a work/life paradox, whereby organizational policies, supervisors, and coworkers attempt to control and shape parents’ boundary enactments and vice versa. *Tensions* reflect clashing ideas or principles and the discomfort that can arise (Stohl & Cheney, 2001). During resocialization, numerous professional and parenting norms emerge as tensioned within the broad assimilation/individualization dialectic. Ultimately, this dialectic illuminates a work/life paradox when parents communicatively navigate competing tensions, including ideal worker and parent norms during resocialization.

Highlighting “the dynamic interplay” between assimilation and individualization (Scott & Myers, 2010, p. 80), we analyze parents’ work/life boundary-setting communication surrounding the tensions made manifest during resocialization after parental leave. Our resulting findings nuance paradox and organizational socialization work by demonstrating how the resocialization process constitutes a site of dialectic struggle between control (organizational assimilation) and resistance (individualization), where a work/life paradox can be alleviated through tensioned, boundary-setting responses. Sensitized by Fairhurst and Putnam’s (2024) call for paradox research to shift away from “paradoxing the dialectic” toward “dialecting the paradox,” we attend to how “paradoxes reflect back on and enable or constrain organizational actions” (p. 14)^2^. Theoretically, we discuss how the boundary-spanning nature of resocialization complicates boundary-setting communication. During resocialization, responding within a work/life paradox demonstrates how interpersonal and structural elements intersect to shape resocialization. Parents’ responses to a work/life paradox highlight practical implications for managing dialectical tensions and recommendations to improve supervisor practices, policies, and team communication during resocialization. In the next sections, we contextualize this work by detailing pertinent scholarship, our methods, findings, and offering a discussion of theoretical and practical insights.

### 1.1. Organizational Socialization

Whether employees enter an organization for the first time or re-enter an organization after temporary leave, they engage in the process of socialization. Organizational socialization involves “the simultaneous interaction of *assimilation*, the efforts of current group members to influence newcomers to meet group needs, and *accommodation*, the efforts of newcomers to influence the group to meet their needs” (i.e., *individualization*; Kramer & Dailey, 2019, p. 97, emphasis added). Assimilation is a “continuing organizational process” over time, experienced by new and experienced employees (Gardner et al., 2022, p. 316). While new organizational members generally assimilate to existing organizational norms (Myers et al., 2025), existing employees who experience planned organizational changes may rely more heavily on individualization which “comprises individuals’ attempts to mold organizations to meet personal needs” (Kramer & Miller, 1999, p. 360).

Planned organizational changes include individual or organizational transitions, such as promotions, mergers, or layoffs, where employees experience changes related to “organizational structures, new rules, new roles, new values, new rewards, and new ways of doing work” (Lewis, 1999, p. 67). Research has explored planned changes which trigger resocialization, including how employees are socialized into a variety of organizational roles (Dailey, 2016), newcomer and incumbent organizational member experiences following change (Gardner et al., 2022), and employee sensemaking following a leader’s exit (Godager et al., 2021). While it is important to understand how employees manage workplace transitions, the present study extends organizational resocialization research in response to Kramer’s (2010) call to demonstrate the boundary spanning nature of organizational socialization by examining resocialization following a work/life event—parental leave. Doing so sheds light on how boundary negotiation strategies shape ongoing role enactments, behaviors, and experiences and lead to practical changes to improve employee and organizational outcomes.

Employees who temporarily leave and re-enter the workplace have a pre-established work identity and may struggle when renegotiating norms and behaviors during resocialization (Hart et al., 2009). For parents, re-entry following parental leave requires significant work/life identity transformation because they “can no longer fully enact their old identity” (Ladge & Greenberg, 2015, p. 980). During resocialization, parents must adjust prior self-concepts into a modified, enduring identity within the context of previously familiar, but now unfamiliar, organizational relationships and structures (Freeney et al., 2022). However, adjusting prior self-concepts becomes complicated when parents confront underlying work/life discourses and norms entrenched in earlier socialization processes.

### 1.2. Work/Life Resocialization Concerns

Organizational structures and culture perpetuated through socialization emerge from ideal worker discourses that shape work expectations and enactments (Blithe & Wolfe, 2016). Ideal worker discourses simultaneously reflect and reinforce what it means to be a fully committed and exemplary employee through societal, organizational, and individual communication patterns (Kirby, 2017). Importantly, these discourses privilege work and professional identities over private life identities, which expose salient work/life tensions during resocialization.

Following parental leave, re-entering the workplace complicates parents’ prior worker identities and enactments (Freeney et al., 2022). While some resocialization experiences emphasize synergies in professional norms and values among employees undergoing planned changes (Singer, 2004), parental leave re-entry experiences can expose a work/life paradox wherein professional/parent ideologies and identities are tensioned. For instance, while organizational policies are designed to support working parents, informal supervisor and coworker communication often reinforces professional norms (Kirby & Buzzanell, 2014), revealing the “difference between the espoused policies and the actual norms” (Kramer, 2010, p. 113).

Because managing and renegotiating work/life obligations are integral during parents’ resocialization, we draw on the following three stressors that illustrate the boundary-spanning nature of organizational socialization: behavior, time, and strain (Kramer, 2010). Extending Kramer’s (2010) boundary-spanning stressor categorizations, we consider how each work/life stressor relates to parents’ communication of work/life tensions rooted in professional and ideal worker norms as follows: (1) behavior stressors are evident through examining work/life *identity enactments*, (2) time stressors are discernable through how employees communicate about work/life *time*, and (3) strain is apparent through work/life boundary *topic spillover* communication.

Professional *identity enactments* include actions such as embracing an ideal worker identity (e.g., saying “yes” to more responsibility, climbing the career ladder; Kirby, 2017), working hard and efficiently (Ganesh & McAllum, 2012), privileging rationality over emotionality (Riforgiate & Sepúlveda, 2021), and being productive, reliable, and highly engaged (Kelly et al., 2010). Professional *time* norms influence how individuals communicate their ideas about and prioritize time at/for work versus private life (Coker et al., 2024a), dictate constant availability to work long hours in and out of the workplace (ter Hoven et al., 2017), and reward proof of time spent working (Kelly et al., 2010). Finally, strain occurring from work/life *topic spillover* relates to professional topic norms that dictate work can spill into life, but life should not intrude into work (Coker & Riforgiate, 2023; Pink & Godager, 2022; Pal et al., 2020). All three professional norms culminate in expected ideal worker identities and enactments which contribute to work/life stressors experienced through socialization processes.

During resocialization, dialectical tensions accentuate a work/life paradox when parents can no longer fully align with professional identity, time, and topic norms that working parents experience differently than employees without children (ter Hoven et al., 2017). Professional norms run counter to deep-rooted societal/Western parenting norms; thus, parents’ resocialization requires navigating tensioned professional-parent norms. For example, the motherhood norm, or belief that women should prioritize motherhood and family care over paid labor (Kirby, 2017), is in tension with the notion of being a good working mother, who effortlessly manages professional and domestic responsibilities simultaneously (Kirby et al., 2016; Turner & Norwood, 2013). Similarly, contemporary social discourses perpetuate paradoxical expectations that fathers be both breadwinners and active, involved parents (de Laat, 2023). While both mothers and fathers experience work/life tensions, mothers are often ascribed the primary caregiver role and in turn experience greater work/life tensions (Kirby, 2017). However, increases in fathers’ parental involvement (Rogers, 2021) alongside gradually shifting societal expectations (de Laat, 2023) warrant studying *both* mothers and fathers. Overall, ideal worker and ideal parent norms involve dedication and full-time commitment, which means working parents often encounter tensions when negotiating professional and parent enactments.

### 1.3. Control and Resistance During Resocialization

Focusing on *the dynamic interplay* between assimilation and individualization efforts provides an opportunity to examine the resocialization process as a tensioned site of struggle where a control/resistance dialectic is made visible through employees’ work/life boundary-setting communication. Fleming and Spicer (2007) defined struggle as “a multidimensional dynamic that animates the *interface* between control and resistance… a process of ongoing, multiple and unpredictable calls (power) and responses (resistance) in which power and resistance are often indistinguishable” (p. 58; emphasis in original). Viewing control and resistance as dialectical recognizes that these processes are interdependent and mutually shaping, occurring together without privileging one or the other (Fairhurst & Putnam, 2024; Zoller & Ban, 2020). A control/resistance lens, where “power and resistance occur in concert,” (Wieland, 2011, p. 165) mirrors the fluid push and pull of assimilation (control) and individualization (resistance) parents navigate during resocialization.

Framing socialization as an organizational effort to control individuals, Kramer (2010) calls scholars to examine how organizational members enact resistance and attempt to alter the organization. Additionally, Wieland (2011) asserts that “resistance needs to be understood within the particular context in which it is enacted” (p. 165). In the context of parents’ resocialization, boundary-setting communication occurs within a control/resistance dialectic. Through this dialectic, resocialization experiences can illustrate how communication facilitates work/life boundary negotiation with reciprocal—but asymmetrical—organizational power relations. Organizational control (assimilation) attempts to shape parents’ professional enactments, and parents’ resistance (individualization) emerges through work/life boundary-setting communication.

The present study also responds to Wieland’s (2011) call to view agency as nuanced, understand how individuals are complicit in their own domination, and examine resistance as both strategic and routine. We extend theorizing on organizational socialization, control/resistance, and paradox by identifying how parents’ work/life boundary-setting communication aligns with and/or resists tensioned professional norms during resocialization. Furthermore, following Fairhurst and Putnam’s (2024) recommendation to approach opposites (e.g., control/resistance, assimilation/individualization, work/life) with paradoxical thinking, we work to identify how parents respond paradoxically to communicate boundaries and navigate tensioned work/life identity, time, and topic stressors. There are numerous responses to organizational tensions (Putnam et al., 2016), including (1) *either-or* approaches, where individuals view dialectical poles as binary and *vacillate* between the two; (2) *both-and* approaches, where dialectics are viewed as interdependent and individuals work to *integrate* dialectical poles; and (3) *more-than* approaches, where individuals attempt to *balance* dialectical tensions through creatively connecting and repositioning them (Putnam et al., 2016; Woo et al., 2017).

Considering the prevalence and importance of parent resocialization alongside work/life boundary-setting stressors, the present study is designed to gain contextual, nuanced insights into how boundary-setting is enacted within a control/resistance dialectic during resocialization. Doing so will deepen theoretical understanding on organizational (re)socialization and paradox theory and illuminate practical implications for organizations, managers, and individuals. Therefore, our study is guided by the following research question: *How do parents communicatively negotiate work/life boundaries within the resocialization control/resistance dialectic following parental leave?*

## 2. Methods

### 2.1. Recruitment, Participants, and Data Collection

To address our research question, we recruited 16 employees who (1) lived and worked in the United States, (2) became first-time parents within the previous two years, (3) took at least one week of paid/unpaid parental leave, (4) worked prior to becoming a parent, and (5) returned to the same workplace following parental leave. Recruiting first-time parents (within the previous two years) allowed us to focus on parents’ resocialization experiences given their new identity as parents (regardless of the number of children participants had during the previous two years).

We specified one-week leave as a criterion to be as inclusive as possible, since many parents cannot afford prolonged unpaid leave. Brown et al. (2020) noted that in 2018, “among employees who reported taking leave in the past 12 months, 42 percent received full pay, 24 percent received partial pay, and 34 percent received no pay while on leave” (p. iv). Even a short leave in conjunction with the significant life event of becoming a parent creates the need for ongoing resocialization.

Recruitment and data collection occurred in the fall of 2023. We limited participants to individuals who became first-time parents in the fall of 2021 or after to ensure participants did not become parents during or directly after the COVID-19 pandemic, as remote work resulted in aberrant/atypical work and parenting experiences (Coker et al., 2024b). It is increasingly common for both mothers and fathers to take parental leave in the United States (Rogers, 2021). To enhance work/life research that overwhelmingly examines mothers’ *or* fathers’ experiences (for exception see Long et al., 2018), we recruited both mothers and fathers. Although mothers and fathers do not take equal amounts of parental leave (Gurchiek, 2019), an abundance of research supports parents being entitled to—and taking—paid parental leave (e.g., Schulte et al., 2017), and parental leave is a goal for many (United States Department of Labor, n.d.). As fathers taking parental leave becomes increasingly commonplace, it is vital to examine the experiences of mothers *and* fathers. Our study takes a step in this direction.

Recruitment occurred as a multiple start snowball sample via social media, personal networks, and participant referrals to others that fit the study criteria (Lindlof & Taylor, 2019). Participants included 14 heterosexual mothers and two heterosexual fathers who were married (*n* = 14) or in a relationship (*n* = 2). Participants ranged in age from 26 to 41 years old (*M* = 32). Most participants were white (*n* = 15), and one chose not to specify their race. Within the previous two years, 13 participants reported having one child, three had two children, and one had twins. On average, participants became parents 12 months prior to their interview and took between 4 and 36 weeks of parental leave (*M* = 13.39). Participants reported varying household income ranges, including $25,000 to $50,000 (*n* = 2), $50,0001 to $75,000 (*n* = 4), $75,001 to $100,000 (*n* = 2), and over $100,000 (*n* = 8). Participants were located across the Midwest and West Coast of the United States, and all interviews were conducted remotely from the state of Wisconsin. See Table 1 for additional participant information.

Sensitized by a feminist approach to in-depth interviewing, we developed an interview guide that allowed participants space to reflect on their most salient experiences and attend to hidden and/or unarticulated experiences (Hesse-Biber, 2014). A feminist approach informed the inclusive interview language, guided our positionality reflections during data interpretation and writing, and helped facilitate a critical examination of parents’ experiences with organizational and societal discourses emerging through their communication. This approach also necessitated that we reflexively and carefully consider how our positionality influenced our data interpretation. Both of us are women with children—one with a small child and one with two adult children. We also both returned to work after taking time off and experienced resocialization in different fields—one in academia and one in the insurance industry. Furthermore, both of us are also work/life scholars steeped in this research and attuned to the many challenges women experience navigating professional and personal identities and demands. As such, we took steps to be attentive to nuances shared by participants while ensuring that we accurately reflected participant experiences rather than our own. This required open conversations about the data, questioning each other, and returning to the data repeatedly to ensure that our findings resonated with participants’ descriptions of their experiences.

Our interview guide began with a substantive, intentional framework of loosely structured interview questions that addressed a consistent set of topics (Hesse-Biber, 2014). This framework helped us understand participants’ experiences becoming a parent, taking parental leave, and transitioning back to work following parental leave (e.g., Tell me: your story of becoming a parent; about your family; details about your parental leave; about returning to work after parental leave). Beginning with broad topical areas allowed us to follow up with tailored probing questions to deeply explore issues/ideas that naturally emerged through participants open-ended responses (e.g., Tell me a story: about a challenge balancing work and parenthood; about a memorable interaction with coworkers/managers when returning to work; that characterizes your transition back to work after parental leave). Addressing consistent topics while tailoring broad and open follow-up questions and probes allowed us to explore participants’ self-identified salient work/life experiences (Hesse-Biber, 2014). We confirmed reaching data saturation when similar sentiments were expressed across multiple interviews (Tracy, 2020). We also engaged in data conferencing with multiple experts to ensure credibility of the findings. Furthermore, to substantiate and support each claim, we included multiple participant quotations throughout the findings section (Tracy, 2020).

### 2.2. Data Analysis

Interviews totaled 17 h and 28 min and ranged from 50 to 90 min (*M* = 65.5; *SD* = 10.2). Interviews were preliminarily transcribed using Otter.ai, then reviewed alongside the original audio for accuracy (Braun & Clarke, 2006). We analyzed 254 single-spaced typed pages of interview transcriptions and 35 single-spaced typed pages of analytic memos that noted initial insights, new questions, and scholarship connections (Charmaz, 2014; Saldaña, 2021; Tracy, 2020).

We conducted a thematic analysis using a flexible and iterative approach sensitized by insights generated from the data and prior research (Braun & Clarke, 2006). The iterative approach involved moving back and forth between the data, relevant research, and theory (Tracy, 2020) to examine nuanced experiences within the control/resistance dialectic during resocialization, while separating and organizing participant actions, feelings, and experiences into categories to develop and substantiate themes. We read the transcripts to familiarize ourselves with the data, noting patterns and interesting elements, and generated 262 initial/primary-cycle codes (Braun & Clarke, 2006; Tracy, 2020). Codes were determined by “assigning words or phrases that capture[d] the essence” of the data (Tracy, 2020, p. 219), including participant actions (e.g., “juggling,” “compartmentalizing”), needs (e.g., “flexibility,” “balance”), constraints (e.g., “expectations,” “culture”), or work/life enactments (e.g., “worker/parent identity,” “setting boundaries”).

We collaboratively sorted and collapsed codes into 44 categories (Saldaña, 2021), which we used as a higher-level codebook when re-reading interview transcripts and re-coding the data (e.g., collapsing “juggling,” “flexibility,” and “compartmentalizing” codes into a “restructuring work to fit life” category). Returning to the data with this codebook allowed for a broader focus and further data organization. Finally, we created tables to (re)organize, (re)visualize, and make sense of emerging themes/sub-themes while considering potential code/category/theme relationships (Braun & Clarke, 2006). During the final analysis stage, we identified representative exemplars of each theme and sub-theme to evidence our findings (Tracy, 2020).

## 3. Results

Parents’ experiences returning to work following parental leave illustrated a control/resistance dialectic situated within an overarching work/life paradox. Tensioned ideal worker (professional) and parent norms shaped participants’ boundary-setting communication; this demonstrated the push and pull between assimilation and individualization during resocialization. Parents’ responses to three boundary-setting stressors—identity enactment, time, and topic spillover—demonstrated how they communicated alignment with professional norms (reinforcing organizational control) and/or prioritized working parent enactments (resisting organizational control). Our findings illustrate how setting boundaries within a work/life paradox can ultimately enable and constrain employee and organizational actions during resocialization in response to identity enactment, time, and topic spillover stressors.

### 3.1. Identity Enactment and Boundary Setting

Workplace reentry following parental leave represented a tensioned experience as participants struggled to (re)negotiate their professional and parent identity boundaries. Within a work/life paradox, participants grappled with tensioned ideal worker and parenting norms. On one hand, responses emphasized maintaining professional identities, and on the other hand, recognized how becoming a parent necessitated reprioritizing worker/parent identities. For example, although Lynn “always thought it’d be nice to be a stay-at-home mom,” she felt “ready to go back to work … I’ve always wanted to make my own money … I went to school for this job … it’s definitely still my own decision to work.” Participants also recognized how their parent identities shifted their values and priorities. Kelly explained,

A massive part of identity is your job. And honestly, since having a kid that is less true for me … I think first of myself as a mom … I can establish a “professional identity,” or whatever, and then at the same time, I’m never not thinking about [my daughter].

While participants referenced previous working identities, parenting forced them to re-evaluate and reconcile new realities of being a worker and a parent. Participants described events that revealed how professional and parent identity boundaries were renegotiated to address resocialization tensions. When reentering the workplace, participants’ tensioned identity boundary-setting enactments emphasized (1) remaining recognized as a professional, rather than “just” a parent, while (2) communicating how a parent identity necessitated changes to their prior professional identity.

#### 3.1.1. Privileging a Professional Identity

Participants found it problematic and resisted when others treated them as “parents” rather than “professionals” at work. Participants’ communication *reinforced their professional identities* by (1) enacting professional norms and (2) ending or redirecting non-work communication. For instance, although Charlotte acknowledged that her “priorities have shifted to family,” she *enacted professional norms* when she “pick[ed] up [work] … that may not be seen by my coworkers,” and recalled being, “a little annoyed that it seemed like folks didn’t want to include me on things.” Charlotte reinforced her professional identity boundary by telling coworkers, “I’m back guys, you can let me know about stuff again … please invite me to the next meeting.”

Similarly, Michelle felt challenged when a coworker contacted her co-lead, “even though the problem was something that I am more knowledgeable about.” Michelle shared her frustration when her coworker made “the comment that, ‘oh, she’s a mother, she’s got a kid at home, I don’t want to bother her.’” Michelle directly confronted her coworker to re-establish professional identity boundaries, emphasizing,

Why wouldn’t you call me when that’s my realm of the lab? … These are two separate things, I can be a mom, and also still be the lead of the lab at work … it’s up to me to decide if you’re interfering with my being a parent.

When coworkers insinuated that parenting overshadowed professional responsibilities, participants’ boundary-setting responses regularly reinforced their professional identity and ideal worker norms (e.g., not allowing life to interfere with work). Doing so illustrated participants’ attempts to control when and how professional and parent identities were foregrounded. By foregrounding professionalism, participants resisted the notion that parenthood diminished their ability to fulfill work responsibilities.

Participants also reflected on how coworkers subtly blurred professional and parent identity boundaries through “chit chat” or “casual conversations” and responded by *ending or redirecting communication* to center their professional identity. Norah recalled conversations that “always go to [my son] … And I love it … But like, there’s part of me that also wants to keep a little bit of separation between home and my work.” She explained why she set professional identity boundaries as follows: “I don’t want to ‘just’ be a mom … I want to still be seen at work as a professional. I want to be seen as more than ‘just a mom who happens to work there.’” Norah described how she often ended “the conversation, like, ‘alright, well, I’m gonna get back to it.’” She also “redirect[ed] a little bit. You know, somebody asks, ‘Oh, how’s baby?’ and I’ll say, ‘Oh, he’s doing great, smiley as ever,’ … then I ask about what’s going on with their life.” Ending and redirecting parent-related conversations allowed Norah to foreground herself as a professional and reinforce work identity boundaries.

During resocialization, participants’ identity boundary-setting communication resisted the idea that being a parent undermined their professionalism by privileging ideal worker identities. They responded to a work/life paradox by vacillating (either-or approach) towards the control dialectic to accentuate professional identity enactments which sidelined parental identities. To affirm their worker identities, participants emphasized their expertise and availability to work while ending or redirecting parent-related conversations.

#### 3.1.2. Creating a Working Parent Identity

Despite setting boundaries by vacillating to foreground a professional identity, participants struggled when discussing the complicated reality of being parents. When a work/life paradox and associated tensions threatened participants’ abilities to fully enact their professional identities, participants attempted to integrate (both-and approach) their worker and parent identities to espouse a working parent identity. Participants illustrated resistance when renegotiating boundary-setting identity enactments by (1) readjusting career goals, (2) making parenting values and priorities clear, and (3) communicating work/life challenges.

Both Kris and Alyssa demonstrated boundary *readjustment around career goals* to privilege a complex working parent identity. Kris, a mother of two children born 14 months apart, strove to maintain her professional identity following her first parental leave by saying “yes” to more responsibility and career advancement, recalling, “I combined teams, and I had double the amount of direct reports and my responsibilities doubled … I didn’t look a gift horse in the mouth. So, I just did it all.” Similarly, pre-parenthood, Alyssa always used to “go to the next step at work or always show that I can do the next thing. And my goal for working was just to try to continue to go up higher in my career.”

However, after Kris’ second parental leave, she was working “all the time, basically, whether taking care of a baby, or working.” In response, Kris began privileging her working parent identity by maintaining her current workload, but not taking on more: “Last year, I purposely did not want that … I had those conversations with career progression where I flat out said, ‘I can’t do more.’” Alyssa also reconstructed identity boundaries by privileging the complexity of her new reality. When Alyssa’s coworkers expected her to work late, she told them, “‘Hey, this can’t happen again’ … There’s going to be a line for me that I’m not willing to cross.” Alyssa fulfilled professional obligations during working hours but instructed coworkers to respect her non-work hours. Kris, Alyssa, and other participants resisted pressures to put career advancement above parenting when resocializing to work. Rather than vacillating between professional and parental identities, they resisted by integrating identities, resulting in work boundaries that privileged the complexity of being a working parent who was not “just” a professional anymore.

Participants also resisted professional norms to integrate a working parent identity by *articulating their parenting values and priorities*. Rob challenged professional norms he identified with prior to parenthood, including “heavy workload[s],” “work[ing] a lot of hours,” and “[being] on call at all hours of the day.” After becoming a parent, Rob explained, “work isn’t important … work isn’t life or death, this doesn’t need to happen right this second. I’m enjoying time with my son.” Rob’s new mindset reset ideal worker/parent norms by redefining the value and urgency of work. Whereas Rob previously aligned with professional norms, including working long hours and responding quickly, he set identity boundaries by integrating tensioned roles, adjusting his values and priorities to attend to work (albeit with less urgency) *and* parenting by crafting a working parent identity.

Finally, participants *communicated work/life challenges* to make parenting demands evident, while simultaneously emphasizing their professionalism to integrate a working parent identity. Many participants used resistance when they “overexplained” to help others understand how being a working parent required redefining professional behavior expectations. Although Charlotte attempted to center her professional identity in coworker interactions, sharing her irritation when she was not included (“with the work I do, it’s good for me to be in on these conversations”), she also complicated her professional identity by making her working parent identity visible to her manager. When discussing her absences after her baby was sick, Charlotte explained how, although she fulfilled professional *expectations*, her professional *enactments* looked different as a working parent:

I just had to sit [my manager] down and say, “This is what we had to do.” … I overexplained. But I think [my manager] needed that … there are just some challenges that, unless you are a parent … it can be hard to understand … I know I’m doing a good job, but I also feel like someone just needs to know our reality. And how hard we’re working to make it work.

Charlotte emphasized her professional identity (“doing a good job”), while resisting the notion that her absences were unprofessional (“working [hard] to make it work”). She engaged in resistance when she “overexplained” so her manager could understand her as a valued working parenting.

Overall, participants illustrated challenges enacting prior professional identities as new parents. Participants’ paradoxical communication responses integrated tensions to reconstruct identity boundaries. Through resistance, they navigated worker/parent tensions by simultaneously marking how they fulfilled reasonable professional obligations while educating others about complex parenting obligations to enact a working parent identity.

### 3.2. Time Boundary Setting

Within the work/life paradox, participants also grappled with the control/resistance dialectic to address time boundary-setting tensions following parental leave. Participants vacillated between (1) intentionally resisting professional time norms and (2) unintentionally reinforcing professional time norms.

#### 3.2.1. Intentionally Resisting Professional Time Norms

Participants described how they intentionally resisted professional time norms that prioritized work by (1) reprioritizing and (2) reallocating their time for parenting. Most participants shared how they *reprioritized* by setting time boundaries to protect private time and fulfill parental responsibilities, resisting professional/ideal worker time norms. Before she had her daughter, Kelly self-described that she was “a workaholic … ‘I don’t need a day off.’ … ‘I love what I do.’ … ‘I’ll work all day.’” However, returning to work, Kelly noted how it “sucked” that her employer demanded constant availability “because it was this expectation like, ‘we know you’d have your email on your phone.’” As a result, Kelly reprioritized her time by setting boundaries that resisted ideal worker time norms of constant availability. She explained, “now I’m like, ‘Nahhh, I think I only want to work six hours today.’ … ‘Bitch, it’s not working hours, leave me alone!’” Similarly, Charlotte’s views on taking time off changed following parental leave. She described, “if I take my sick time, if I use my vacation, that’s just … mine, and I don’t necessarily need to explain myself.” Many participants experiencing time tensions vacillated by resisting professional norms to reprioritize personal time boundaries.

Additionally, participants reflected on how parenthood instigated perpetual time boundary adjustments that necessitated *reallocating their time for parenting*. Stacia illustrated how professional availability norms shaped her time flexibility before parenthood as follows: “Prior to going on leave, I supported between six and eight salespeople … I used to lay out ‘rules of engagement’ … you know, ‘don’t put anything on my calendar before 8am’ … And I would make exceptions all the time.” After becoming a parent, pumping breastmilk at work required Stacia to re-evaluate her time flexibility and provided her with ongoing means to challenge availability norms. Stacia explained,

[The salespeople] had no choice [but to respect my calendar]. I had set times [to pump] … I’m like, “you want me to do a demo during my lunch? Well, I’m not doing a demo with my shirt off, so I’m going to pump in peace.” … The fact that I had something that I *had* to do, … my body would have a physical reaction if I didn’t do it, helped keep that boundary. But even after I stopped pumping, just having had those months of practice in holding that boundary firm now helps me continue that.

Other mothers resisted professional time norms by reallocating time boundaries around embodied elements of parenting, including breastfeeding, pumping, and Cesarean-section recovery.

Furthermore, both fathers and mothers discussed how external parenting responsibilities helped them enact strict professional time boundaries. Childcare constraints were commonly used to justify setting well-defined time boundaries. Rob described how for routine “daycare” or “emergency, immediate matters,” he “would just drop everything and go … work isn’t preventing me from being able to do that.” Similarly, Anne was “very clear … like, ‘listen, at four o’clock I have to leave because I have to go pick up my kids.’” Participants’ intentional time boundary resistance was shaped and justified by physical necessities and external organizational constraints such as daycare operating hours. They vacillated to honor their parenting responsibilities by pushing against previous professional time expectations.

#### 3.2.2. Implicitly Reinforcing Professional Time Norms

Despite intentionally communicating private time boundaries, many participants perceived pressure from coworkers and/or managers to uphold professional time norms. Participants managed this tension by outwardly resisting but *implicitly conforming to uphold professional norms* associated with visible work time, productivity, and organizational commitment. Participants’ desire to resist professional time norms but overall failure to enact time boundaries illustrates the push and pull of organizational and individual agency during resocialization. Participants’ behaviors implicitly reinforced professional norms when they (1) worked additional time outside of work hours, (2) worked more efficiently, and (3) made work visible to substantiate work time.

Participants regularly described setting flexible time boundaries around work hours. On the surface, this helped them resist professional time norms of being physically present during working hours. However, participants inadvertently reinforced professional norms by *working additional time outside of traditional work hours*. Although Kris took advantage of flexible work time boundaries for daycare pick up, “it wasn’t an option to say no” to the number of working hours. Kris would “go pick up, I’d come home, I’d kind of be working and making dinner and doing all the things. Or I would just wait to put them to bed and then go back to work.” Similarly, Anne worked additional hours from home to accommodate her work and parenting responsibilities. She explained, “I actually get up pretty early … do a little bit of work … checking emails, checking meetings … then do my mom stuff … and then get back into work.” In these cases, participants conformed to—and in some cases, amplified—professional time norms by working *more* total hours in and outside of the office compared to before their parental leave.

Alternatively, by setting strict time boundaries, some participants relinquished worktime flexibility they previously took advantage of and *worked more efficiently* to increase productivity. Norah explained, “[I was] a completely different worker before [becoming a parent] … I used to work much more leisurely … if I got sucked into a social media rabbit hole … or if I decided to take a bath in the middle of the day … that was fine.” After her parental leave, Norah’s work time boundaries became increasingly rigid:

As soon as I get in the door from dropping [my son] off at daycare, I’m frantically on my computer—typing, typing, typing—whatever I need to do until the moment I leave to go pick him up … It’s just a lot of, like, being uber-productive … not even taking lunch breaks or anything like that.

Participants’ strict time boundaries aligned with professional norms of high productivity, efficiency, and time allocated for work above time for life.

Participants also aligned with professional norms by *making work visible to substantiate work time*. Although Charlotte resisted professional norms, claiming her time off was hers and she did not need to provide explanations, she also shared,

I don’t think my coworkers realized I got [to work] at seven o’clock in the morning and I didn’t have a lunch break. Or I was working at night because that’s not necessarily something you see … I did explain, “We don’t have care for my son after 2:30pm, so I’m up every morning, often I’m working at night.”

Despite intentionally resisting by prioritizing time off, Charlotte implicitly reinforced professional norms by making invisible morning and evening work time visible:

When I would put in a couple hours at night, I’d always try to make sure there was something with a paper trail … sending an email so that the work was seen … I was like, “Well, I want to make sure that they know I am doing things.” … Everybody else had more normal schedules, I just felt like I needed to make my work seem “more.”

By making work apparent through explicitly describing and leaving email paper trails, Charlotte vacillated between fulfilling professional time expectations while intentionally taking advantage of flexible time for parenting, demonstrating a complex control/resistance dialectic.

Considering the broad work/life paradox that manifested through control/resistance dialectics, participants had difficulty integrating time roles and instead vacillated between resistance (allocating parenting time) and control (allocating work time). Time was described as a finite resource where work/life responsibilities were perpetually in competition, indicating a strong push and pull between dialectics. Ultimately, participants intentionally embraced flexibility and parenting time boundaries but implicitly vacillated towards professional norms by working additional time outside of work hours, working more efficiently, and making work visible to substantiate work time.

### 3.3. Topic Boundary Setting

Within the work/life paradox, participants’ topic boundaries also constituted a dialectical control/resistance struggle, where communication responses enabled participants to navigate tensioned topic norms by (1) limiting private topic spillover to reinforce professional norms and (2) resisting professional topic boundaries to balance work/life experiences.

#### 3.3.1. Reinforcing Professional Topic Norms

Participants recognized professional topic norms that dictate private life should not intrude into the workplace; this constrained their ability to comfortably enact both worker and parent roles following parental leave. Boundary topic setting included (1) withholding parent-related topics at work and (2) only discussing personal/parenting topics out of professional necessity. Separating work and life topic boundaries by *withholding parent related topics at work* allowed participants to privately enact parent roles but publicly fulfill professional roles. Michelle explained that there are private, parent-related topics that “you would probably not say out loud” at work. After returning to work, Michelle reflected on instances when coworkers asked if she was pregnant again or planning to have another baby. Michelle remarked that it was “such a line that was crossed.” She only felt comfortable discussing topics at work that allowed her to be seen “as a professional [rather] than a mother.”

Similarly, Blair reinforced professional topic norms noting that discussing parent-related topics at work “overstep[s] boundaries” and “blur[s] the line between business and personal.” Blair “give[s] less information” about her personal life and explained, “If I want to take my daughter to the zoo for animal class … I don’t have to tell them … I’ve started being like, ‘I have an appointment, and I have to leave.’” Blair’s conformity to professional norms by limiting personal topics and disguising plans with her daughter as “an appointment,” draws on professional language to prevent topic spillover while allowing her to discretely prioritize parenting. Blair further reinforced professional norms in reaction to her boss’ topic spillover:

[My boss] thinks that he just knows everything about parenting … he’ll try to give me “big brother” advice … Like, “I don’t need you to tell me how to parent … it doesn’t pertain to work. So why are we talking about it?”

In both cases, Blair vacillated to enact control by disciplining herself *and* others to reinforce professional topic boundaries.

Interestingly, Kris encouraged her direct reports to discuss parenting topics at work, but felt it was unprofessional for her to do the same. Kris described being “pretty vocal” about wanting to “change” parental leave policies and flexibility to put “people first” in her organization. She said, “it’s really important for me to lead in a different capacity, and share that … ‘it’s okay, take the time that you need.’” However, Kris reinforced professional norms by hesitating to share her pregnancy information: “I just don’t feel that [putting people first] includes me … I really didn’t feel that way when I was having babies back-to-back. It almost felt embarrassing to tell them I was pregnant again.” Kris provides a powerful example illustrating how she vacillated to control her own work/life topic boundaries, which contradicted her encouragement of others to resist to integrate working parent boundaries.

Many participants only *discussed personal/parenting topics out of necessity*. In this way, participants conformed with professional norms by making a rational business case for sharing personal information. For instance, Blair “didn’t even want to share” her traumatic labor and unplanned Cesarean-section with her managers, but “felt like I had to because I had to make the case of like, ‘I can’t physically work as much as you want me to.’” Ultimately, Blair felt “cornered into telling them all this stuff … that was more personal than I would like to get with them in order to keep my job.” Her hesitation to share physical limitations so she could continue working demonstrates her perception that topic spillover is only appropriate based on business necessity. Taken together, participants vacillated toward a control dialectic by limiting and policing their own and others’ personal topic spillover, which constrained resistance, further perpetuating professional norms. However, as participants maintained professional topic norms, at times they creatively used professional language to meet personal needs or goals (e.g., camouflaging parenting “appointments”, being vocal about putting “people first” as a manager) offering a hint at resistance working within the control dialectic.

#### 3.3.2. Resisting Professional Topic Norms

Some participants also resisted professional topic spillover norms by (1) infusing their working parent experiences into the workplace and (2) framing work/life topic spillover as an opportunity to challenge professional norms to enhance others’ experiences. First, parenthood allowed some participants to better understand and be more compassionate by *infusing their working parent experiences into the workplace*. Lynn explained how becoming a parent shifted her perceptions as follows: “When people talked about their families before … I didn’t feel like listening. But now … I feel like I understand them more than I did before I had a child.” Similarly, Stacia reflected, “I have a different level of understanding … I engage with [colleagues] differently … it has given me more ability to just take different perspectives and have deeper empathy with a wider range of circumstances.” Kris shared that becoming a parent enhanced her workplace interactions: “I am so serious at work … [but when] I talk about my kids at work, it softens me … it brightens my face, and it gives me something that’s a little easier to relate to.” Blurring topic boundaries made her more compassionate and relatable as a manager. These participants went beyond vacillating or integrating, by balancing (more-than approach), noting how parenthood made them better workers and/or organizational leaders. These responses illustrate how participants held the tensioned ideal worker/parent poles together to create a new way of thinking within the work/life paradox by transforming topic tensions into productive, ongoing actions.

Furthermore, at times participants *framed work life spillover as an opportunity to challenge professional norms to enhance other parents’ experiences*. Doing so allowed participants to envision ways to positively shape their leadership. Kris explained how she “didn’t feel [supported as a parent], so it’s really important for me to lead in a different capacity and share, ‘it’s okay, take the time that you need.’” She elaborated, “My goal is to make it easier. … I want it to be a better situation and experience for the next level, or we won’t be able to continue to hire younger people who want to have families.”

Stacia resisted professional norms by embracing blurred work/life topic boundaries, “encouraging people” and “proactively telling [other parents] to take more breaks or to have more grace.” She explained,

[My direct reports are] like, “we have to work,” and it’s like, “your kid is home sick with hand, foot, mouth [disease]. Please, do not jump on calls. Our work is not that important” … I honestly just don’t think I would have taken that approach before. … You strive to be empathetic, but unless you really have [parenting experience] to pull on, it’s very difficult to fully understand.

Participants’ balancing of work/life topic tensions demonstrated how they connected dialectics through communicating that parenting enhances work. Doing so allowed them to take steps to ameliorate tensions.

Overall, in both reinforcing and resisting, participants’ boundary-setting topic responses helped them maintain personal control. When participants conformed to professional norms by avoiding topic spillover, they maintained clear topic boundaries to protect their private life. When participants resisted professional norms, they allowed topic spillover between their working parent and professional roles in ways that benefited their own and others’ work/life management. Next, we discuss the theoretical and practical implications of our findings.

## 4. Discussion

We sought to understand how parents communicatively negotiate work/life boundaries within the resocialization control/resistance dialectic following parental leave. Our findings provide a nuanced and contextual (Wieland, 2011) understanding of the ways boundary-setting stressors (Kramer, 2010) varied in complex and interwoven ways as parents resocialized to work following parental leave. Participants’ identity enactment communication vacillated to privilege a professional identity at times, particularly when threats occurred to worker identities. Other times participants integrated to form a working parent identity by reinforcing they were competent workers and needed others to understand their new parental reality. Participants vacillated regarding time stressors, on one hand intentionally resisting ideal worker norms to privilege parenting priorities, and on the other hand unintentionally reinforcing ideal worker norms. Finally, participants vacillated to address topic stressors by limiting their own and others’ parenting communication to reinforce professionalism. Yet, even when self-censoring occurred, several participants used a balancing approach to enhance supervisory relationships and reduce tensions for others. These findings reinforce that resocialization complicates prior worker identities and enactments (Freeney et al., 2022; Millward, 2006).

### 4.1. Theoretical Implications

Theoretically, our study has numerous implications for organizational paradox scholarship (Fairhurst & Putnam, 2024) and the model of organizational socialization in the resocialization phase (Kramer, 2010). We answer Fairhurst and Putnam’s (2024) call for “dialecting the paradox” and advance paradox metatheory by “examin[ing] how paradox and dialectical tensions are reciprocally interrelated” (p. 184). First, our findings evidence how dialecting the paradox during resocialization is a process where paradoxical responses to tensions “open up a space for action” (Fairhurst & Putnam, 2024, p. 185). Participants’ resocialization communication around identity enactment, time, and topic boundary-setting stressors exemplified how control/resistance dialectics were interwoven, complicated, and evolving. This complexity illustrated competing tensions that challenged ideal worker and parent norms contributing to and emanating from a work/life paradox. Dialecting a work/life paradox illuminates how this paradox was performed in practice during resocialization as a *process* rather than a static dilemma. The paradox emerged alongside tensions and was performed through participants’ enactments of control (assimilation) *and* resistance (individualization) as they kept ideal worker and parent norms in play by vacillating, integrating, and/or balancing these tensions via tensioned boundary-setting enactments.

Second, our study illustrates how a work/life paradox is a communication phenomenon generated throughout individual, organizational, and societal discourse levels and demonstrates how “paradox has organizing potential in constituting the organization” (Fairhurst & Putnam, 2024, p. 3). In dialecting a work/life paradox, we found evidence that the paradox extended beyond organizational control to societal discourses that were reinforced and/or rejected through individual actions. These findings also nuance prior work/life scholarship that suggests it is supervisors’ and coworkers’ communication that reinforces professional norms (Kirby & Buzzanell, 2014; Kirby & Krone, 2002) by recognizing additional constituting influences.

Participants’ boundary-setting communication was shaped by organizational norms and societal ideal worker and parent discourses. For example, participants demonstrated their commitment and professionalism by rejecting others’ foregrounding of their parent identities, working longer hours outside work, and avoiding parenting topics. Together, a work/life paradox was (re)constituted when individual-, organizational-, and societal-level discourses merged within and influenced participants’ boundary-setting communication. These discourses reciprocally enabled and constrained organization of a work/life paradox and what was possible through ever-evolving interactions across discourse levels. Thus, our study confirms prior scholarship that demonstrates organizations are rife with tensions and paradoxes (Fairhurst & Putnam, 2024). We extend paradox work by demonstrating how the experience of a work/life paradox during resocialization was a communicative phenomenon, shaped by individual, organizational, and societal discourses that necessitated constant tension negotiations across boundary-setting stressors.

Third, answering Kramer’s (2010) call, our findings demonstrate the boundary spanning nature of organizational socialization, both physically (i.e., workplace, home) and discursively. We also add to the model of organizational socialization (Kramer, 2010) by illustrating how the resocialization process is reciprocally shaped by/through paradox. Resocialization and paradox are simultaneously negotiated through communication, at multiple discourse levels, and generate individual and organizational change. Participants’ tensioned responses illustrated how resocialization facilitated creative maneuvers within a paradox, such as outwardly aligning with organizational discourses valuing work above life, while also leveraging professionalism to resist prioritizing work above life. For example, Blair adopted professional language, calling time with her daughter “appointments” to leverage professional topic norms to protect her private time. This study enhances understanding of resocialization as a paradoxical experience where tensions emerge and can be creatively reinterpreted or reconfigured through employees’ being complicit in control (assimilation) and/or engaging in resistance (individualization). Within these control/resistance processes, paradoxical responses to tensions can allow individuals to maneuver between assimilation and individualization in ways that help them feel in control across work/life boundaries.

Fourth, by demonstrating participants’ tension engagements during resocialization, our findings highlight how keeping tensions in play via paradoxical responses, as opposed to simply labeling or attempting to resolve tensions (Fairhurst & Putnam, 2024), enables and constrains individual-level boundary-setting communication that drives organizational action. While resocialization requires navigating the control/resistance dialectic, findings suggest that the experience of a work/life paradox and the tensions that maintain it can disrupt or recontextualize prior work understandings and enactments. For example, Rob’s experience of a work/life paradox disrupted his prior worker mindset and re-established his time boundaries and values. Boundary setting during resocialization allowed participants to enact new conceptualizations of identity, (re)adjust enactments of time, and develop different ways to conceptualize work/life topics in alignment with or resistance to professional norms.

Therefore, within the overarching perspective of paradox, resocialization following a temporary leave provided participants with an opportunity to hold “opposite [tensioned] poles together,” keeping dialectical tensions in play and maneuvering within them to “gain agency and use paradoxical performances to transform situations” (Fairhurst & Putnam, 2024, p. 185). For example, participants’ resistance to professional topic norms transformed into a business case for why changes were needed to attract and retain employees (e.g., “…or we won’t be able to continue to hire younger people who want to have families”). Furthermore, physical (e.g., pumping) and external time demands (e.g., daycare hours) prompted resistance as tangible constraints to justify change and advocate for a workable solution. Additionally, while participants were not always comfortable advocating for themselves, many used their supervisory position and power to create forward change. Our findings provide examples of how “paradoxical performances in response to organizational paradoxes may be an effective way to find room to maneuver in the midst of irreconcilable alternatives” and where work/life tensions and stressors could be held together to “provide an opening for alternative action” (Fairhurst & Putnam, 2024, p. 186). Ultimately, participants’ communication approaches to boundary-setting tensions support Wieland’s (2011) argument that agency is nuanced. Our findings provide tangible examples of how resistance can be routine and strategic during resocialization.

### 4.2. Practical Implications

Organizational members’ needs change over time (Hart et al., 2009) and resocialization is an integral part of that change process. Our study focused on resocialization after parental leave but there are numerous life events, including medical issues and caring for family members, that generate dialectical tensions and a work/life paradox. For example, caring for an elderly parent can call into question worker and parent/child roles, requiring identity enactment renegotiation, time adjustments, and topic boundary attention. Thus, while our study is specific to parents’ resocialization following leave, our findings can be more broadly applied to common life events that trigger temporary leaves and organizational re-entry.

Our practical implications emphasize ways organizations, managers, and individuals can shift dialectic struggles away from individual issues to create more sustainable solutions for organizations and their members. Identity enactment, time, and topic boundary-setting stressors challenged parents reentering the workplace. Participants’ stories and responses indicated they felt personally responsible for managing tensions and, for the most part, keeping life outside of work. This is not surprising; childbirth and parenting have been framed as individual and private issues reinforced across organizational and national policies (Schulte, 2014), which helps explain the difficulties participants experienced in resocializing. Individual responses reduced participants’ ability to address organizational constraints and develop beneficial solutions for themselves and others. Considering that nearly all participants were in white collar roles, predominantly had paid leave benefits, and had some work flexibility yet struggled, points to the even greater challenges workers in more precarious roles likely face within a work/life paradox.

Emanating from our findings, we offer adjustment starting points for each stressor. Our recommendations are informed by Fairhurst and Putnam’s (2024) assertion that “creating paradoxical performances to respond to paradox then disrupts, challenges order, introduces disequilibrium, and provides an opening for alternative actions” (p. 186). First, identity enactment stressors can be ameliorated by training supervisors and organizational leaders to recognize and regularly communicate how they value the professional contributions of employees to affirm this identity anchor (Johnson et al., 2023), particularly for parents reentering the organization. Professional identity affirmation can easily be embedded into organizational culture through discursive comments and actions via organizational leaders, managers, and employees. Recognizing and attending to workers’ self-presentation identity goals increases communication competence and enhances conflict/problem solving communication (Canary & Lakey, 2013). Furthermore, employees can make the demands of being a working parent visible by clearly articulating shifting work/life values and priorities while simultaneously communicating how they plan to fulfill work expectations through new enactments of work. We acknowledge that encouraging professional identity affirmation can reinforce ideal worker norms. However, if done well and sincerely over time, affirmations build trust and identity safety which can facilitate mutual problem solving to support complex work/life identities and hold tensioned identities together.

Time boundaries also present an opportunity. Participants felt pressured to work outside of traditional hours, work longer hours, and substantiate their work. For example, Kris enacted strict time boundaries to pick up her children from daycare but worked additional evening hours when her children went to bed. Similarly, Charlotte worked early in the morning and left “paper trails” to evidence work time beyond business hours. It is important to acknowledge that in practice, one-size-fits-all work/life policies geared toward time flexibility may not alleviate the tensions and pressures working parents experience (Kirby & Krone, 2002). However, to reduce time boundary tensions, supervisors can set reasonable work expectations, focus on outcomes/results (rather than hours working), and make employees’ contributions visible. For instance, a shared digital “to-do” list with task owners can streamline coordination and allow team members to visibly record and communicate work completion. Another strategy is to hold team meetings to solicit employee input on when work is completed (e.g., flexibly) and how expectations are being met (e.g., non-traditionally, but nonetheless satisfactorily) to ensure employees’ voices and contributions are recognized. Open discussions surrounding work expectations can mitigate concerns that work time and efforts are unseen. Creating new workflows and expectations around outputs can disrupt and challenge the tensions generated by sedimented time norms.

Additionally, our findings illustrate how paradoxical performances surrounding tensioned topic norms can transform how parents are perceived in the workplace. For example, Stacia’s experiences with workplace re-entry changed the way she treated other working parents when life concerns (e.g., a sick child) interfered with work. Interestingly, participants in leadership positions held themselves accountable to ideal worker norms yet tried to change the organizations through balancing topic stressors. Kris specifically noted that she was concerned the organization would lose access to good workers if change did not occur. As resocialization is a period of adjustment that can reshape organizational culture (Kramer, 2010), working parents—most importantly those in leadership and managerial roles—should take advantage of opportunities to share new insights and perspectives gained from their experiences, especially as they relate to empathy and an increased understandings of others’ experiences and needs, to reshape organizational culture and improve parental leave and resocialization experiences for others.

Finally, recognizing that every organization is nuanced and contextual (Wieland, 2011), there is not a one-size-fits-all recommendation for workplaces. Organizations and their members could benefit from seeking feedback from working parents either through anonymous surveys or focus groups led by outside consultants as to the structural and relational needs of working parents. Our study participants negotiated each boundary-setting stressor differently which made a cohesive communicative approach challenging. However, identifying dialectical tensions and the paradoxes they generate can facilitate opportunities to engage in paradoxical performances and create space for organizational action and change.

## 5. Limitations and Directions for Future Research

Although this study intentionally explored mothers’ and fathers’ work/life negotiations during organizational resocialization, most participants were mothers. While fathers used similar strategies to navigate the work/life paradox as mothers in our study, future research should solicit additional experiences from mothers, fathers, and adoptive parents to explore differences pertaining to boundary setting and paradoxical responses. For example, some identity enactments might illuminate gender(ed) norms (Kirby, 2017), the emotional labor of the renegotiation of work/life boundaries, or how embodied parenting elements shape boundary-setting enactments. Additionally, it is important to recognize that mothers and fathers likely experience resocialization differently. Due to persistent ideological assumptions that mothers prioritize children over work while fathers are granted a “breadwinner” status—and therefore some leeway for parenting (Buzzanell et al., 2005)—future research should further examine participants’ identity enactment, time, and topic stressors as their communication is shaped by complex, gendered ideological norms.

Additionally, this study focused on a single-country sample (United States) and captured the experiences of 16 educated, privileged, and predominantly white professionals, as all but one participant had the financial ability to choose whether to return to work. However, we recognize that country of residence, socioeconomic status, and class heavily influence the amount of parental leave individuals can take (particularly unpaid leave) and the necessity to return to work. Therefore, we encourage future studies to critically analyze resocialization experiences of workers in relation to location, income, gender roles, and class to examine the work/life renegotiations of employees in cross-cultural contexts and/or parents that do not have a choice to work. In particular, depending on the cultural context, fathers may be penalized or lauded more for caregiving work, which may necessitate different strategies depending on how their behaviors are interpreted within that culture. Exploring gendered parental expectations alongside tensioned boundary management strategies within and across cultural contexts will ensure practical recommendations are appropriate for ideal worker and parenting norms specific to the context.

Future research must also attend to how work/life negotiations during organizational resocialization may vary across diverse, intersectional identities (e.g., race, sexual orientation) to enhance understandings of different cultural or familial expectations. Race can influence cultural expectations of family assistance (more or less) with childcare and impact resocialization. Research including greater diversity and intersectional work would increase understandings of parental leave and work/life interactions and paradoxes.

In the same vein, our study included a mix of remote, hybrid, and onsite workers, as well as participants who did and did not hold supervisory roles, which may have blurred findings related to how participants experienced and responded to identity enactment, time, and topic boundary-setting stressors, dialectical tensions, and a work/life paradox. Especially given the attention to remote work and parenting following COVID-19, future research should examine and compare a broader configuration of employee work arrangements and roles to determine how these factors shape resocialization experiences. For example, in many cases remote or hybrid work provides greater autonomy, which may reduce some boundary tensions while exacerbating others; workers may feel less need to control identity and topic boundaries because interactions can be edited through email and instant messages (Shin et al., 2025), but feel a greater need to justify their productivity and contributions when managing time boundary tensions (i.e., creating paper trails; justifying work hours).

Finally, our study focused on identifying how parents communicatively negotiated work/life boundaries within the resocialization control/resistance dialectic. We identified how parents vacillated, integrated, and balanced their identity, time, and topic boundaries. However, participants did not consistently and uniformly use one strategy across boundaries. While confidentiality agreements may prevent data sharing and replication, our findings can inform future research considering how strategy frequency within each of the boundary contexts influences employees’ organizational identification, commitment, and citizenship behaviors among other variables.

## 6. Conclusions

Overall, this study addressed the following research question: how do parents communicatively negotiate work/life boundaries within the resocialization control/resistance dialectic following parental leave. Our findings answer this question by demonstrating how workplace resocialization following parental leave requires an ongoing negotiation of control/resistance dialectical tensions within a work/life paradox. Participants’ tensioned enactments illustrated dialectic tensions across identity, time, and topic boundary-setting stressors as parents vacillated, integrated, and/or balanced between ideal-worker/professional norms and/or working parent identities. The interplay between dialectical tensions during resocialization at individual, organizational, and societal levels demonstrates the complexity of navigating a work/life paradox and how this paradox creates opportunities for uniting tensions and communicating new possibilities. The study findings extend paradox theorizing and scholarship by (1) illustrating the ways paradoxical responses to boundary-spanning tensions create opportunities for change, (2) establishing how organizational paradox is a communication phenomenon, and (3) explaining how keeping tensions in play enables and constrains individual boundary-setting communication that drives organizational action. This study also theoretically contributes to the model of organizational socialization by demonstrating how (re)socialization communication processes are boundary spanning. Furthermore, we offer practical strategies for managing identity, time, and topic boundary tensions that can be tangibly implemented. Organizations, managers, and individuals should use these strategies to recognize and implement communication practices to address resocialization tensions, ameliorate work/life challenges, and improve work experiences.

## Figures and Tables

**Table 1:** Participant demographics.

Pseudonym	Age	Number of Children	Weeks of Parental Leave	Position	Industry	Work Arrangement	Supervisory Role
Alaina	34	1	12	Pediatric Nurse; Clinical Enhancements and Quality Clinician	Health care practitioner and technical	Remote	No
Alyssa	27	1	13	Senior Financial Analyst	Production	Hybrid	No
Anne	41	2 (twins)	16	Media & Reputation Consultant	Business and Financial	Remote	No
Blair	26	1	4	Designer/Estimator	Installation, Maintenance, and Repair	On site	Yes
Charlotte	30	1	16	Assistant Director of Admissions	Education, Training, and Library	On site	No
Kelly	35	1	12	Psychotherapist; Doctoral Student	Health Care Practitioner and Technical	On site	No
Kris	41	2	9; 10	Director, Talent Acquisition	Management	Remote	Yes
Fiona	29	1	12	Billing Supervisor	Sales and Related	Hybrid	Yes
Leslie	35	2	12; 12	Teacher’s Aid	Education, Training, and Library	On site	No
Lynn	28	1	12	Registered Nurse	Health Care Support	On site	No
Michelle	28	1	12	Lead Medical Laboratory Scientist	Health Care Practitioner and Technical	On site	No
Norah	32	1	36	Assistant Professor	Education, Training, and Library	Hybrid	No
Paige	31	1	12	Registered Nurse; Lead Support Technician	Health Care Support	On site	No
Rob	26	1	6	Application Engineer	Computer and Mathematical	Remote	No
Stacia	35	1	28	Lead Solution Engineer; Team Lead	Sales and Related	Remote	Yes
Zachary	31	1	16	Information Technology	Business and Financial	Hybrid	No

## Data Availability

The data presented in this article are not readily available because the data are part of an ongoing study and the participant consent form did not indicate that data would be distributed beyond study publications.

## Notes

1 For example, ideal worker and ideal parent norms perpetuate the belief that one must demonstrate complete dedication to the respective sphere, where ideal workers invest all their time and energy into work and ideal parents focus their efforts exclusively on investing in childrearing (Kirby, 2017).

2 Earlier work on paradox and dialectics drew on “existing paradoxes to study dialectical tensions” (Fairhurst & Putnam, 2024, p. 14). Fairhurst and Putnam’s (2024) call to engage in “dialecting the paradox” contributes toward a broader understanding of how dialectical tensions generate paradoxes, which enable and constrain organizational actions.
